# Supplementary figures and images for: Systematic mapping of rRNA 2’-O methylation during frog development and involvement of the methyltransferase Fibrillarin in eye and craniofacial development in Xenopus laevis
Source: PLoS Genet. 2022 Jan 18;18(1):e1010012. doi: 10.1371/journal.pgen.1010012 (PMC8797249; doi:10.1371/journal.pgen.1010012)

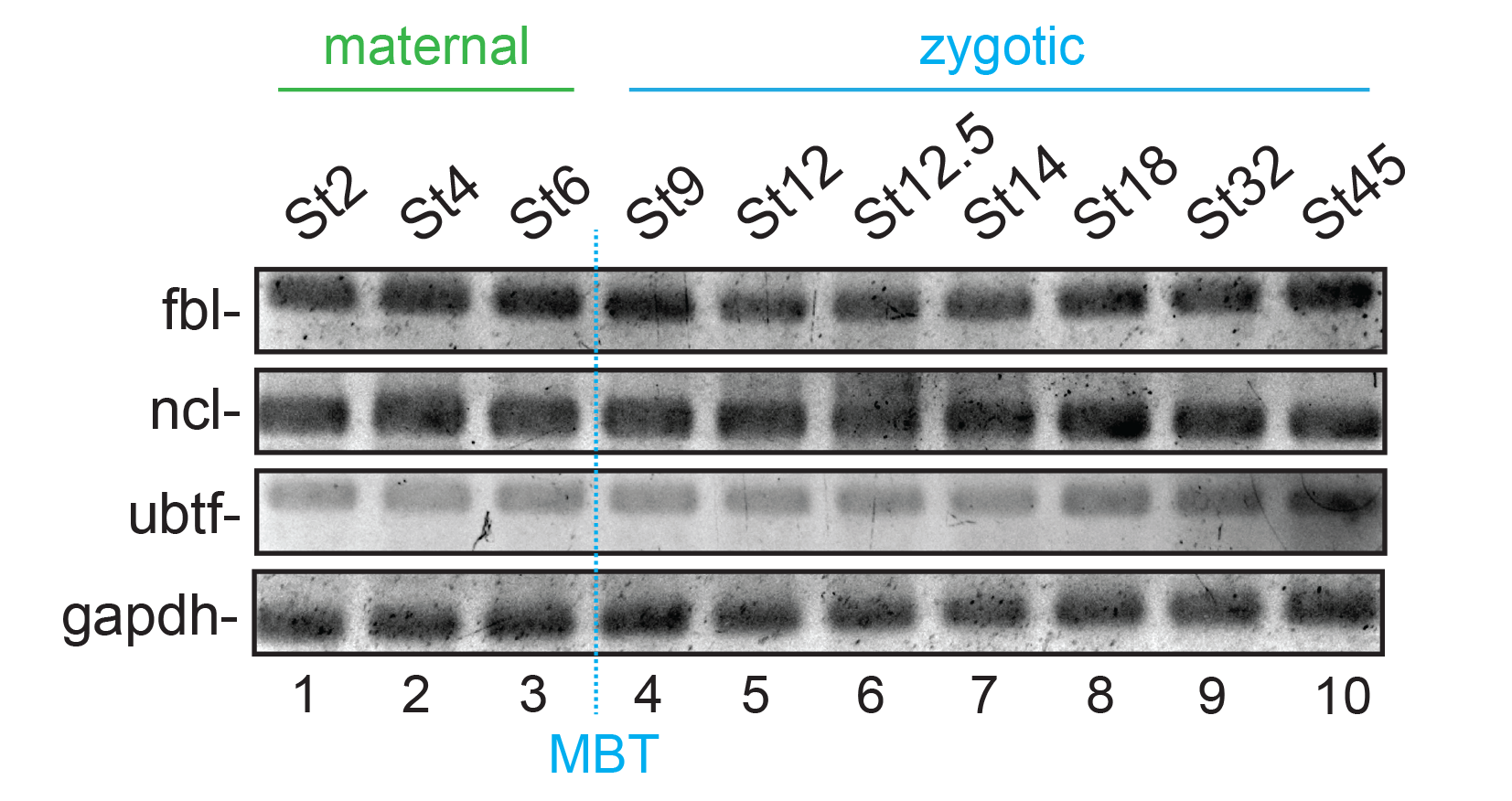

Supplement: S1 Fig — Total RNA extracted from embryos at the indicated stages was analyzed by RT-PCR using amplicons specific to fbl, ubtf, or ncl transcripts (see Materials and Methods). (TIF) [file pgen.1010012.s001.tif]

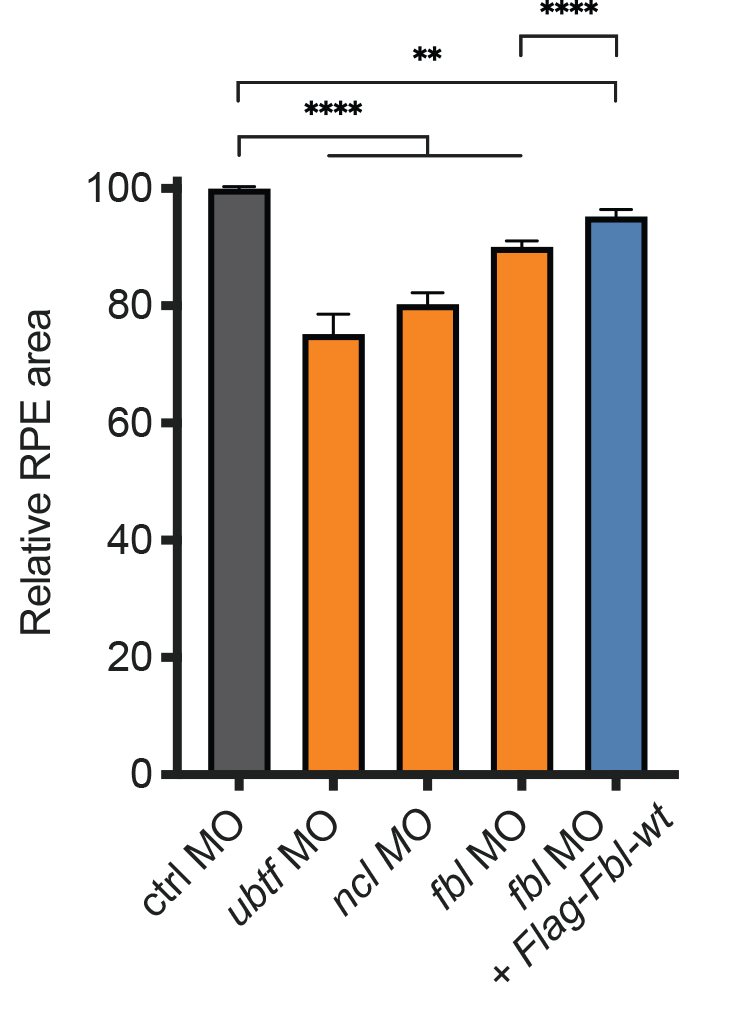

Supplement: S2 Fig — Relative retinal pigmented epithelium (RPE) area (Kolmogorov-Smirnov test, **** = p<0.0001, ** = p<0.01). (TIF) [file pgen.1010012.s002.tif]

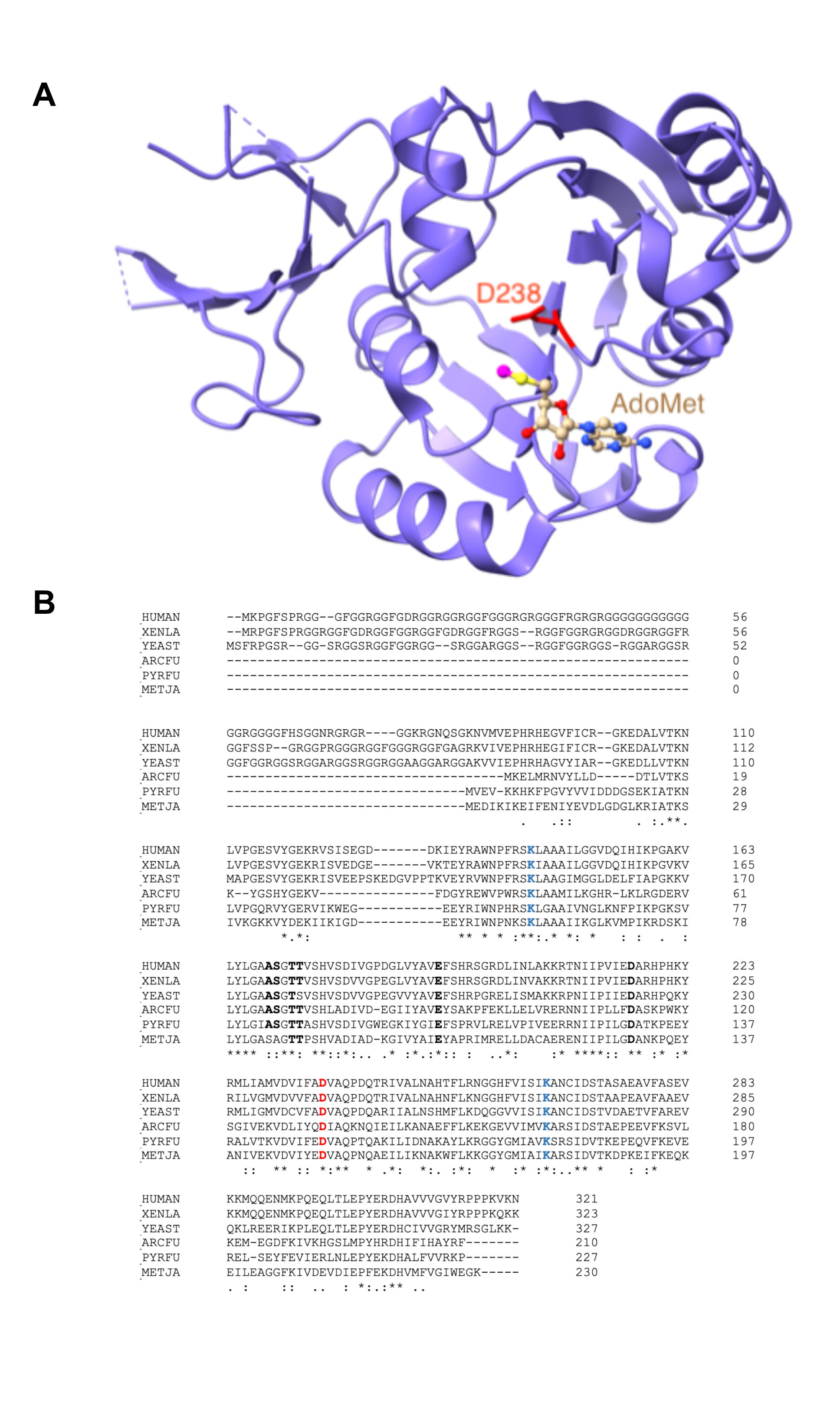

Supplement: S3 Fig — The residue mutated in this work (D238 in Xenopus laevis) is highlighted in red in both panels. In the atomic resolution structure of Archaeoglobus fulgidus fibrillarin-Nop5 complex bound to its cofactor and methyl donor S-adenosyl-L-methionine (AdoMet), it was shown that Asp-133 (equivalent to Xenopus laevis D238) is situated within 3.5 Å of the thiomethyl carbon of the bound AdoMet, implying that it plays a role as a catalytic residue [48]. When this residue was mutated to an alanine, the methylation activity of the complex was indeed totally abolished in an in vitro methylation assay [49]. It has been suggested that Asp-133 in fibrillarin may act as a general base by deprotonating the 2’-OH group of the target RNA during catalysis. It has further been suggested that Asp-133 may also facilitate cofactor binding through favorable electrostatic interactions [48,49]. A, 3-D model of the catalytic pocket of human fibrillarin (based on PDB 2ipx). D238 (in red, Xenopus numbering) is directly adjacent to the AdoMet (stick representation) with the methyl group to be transferred from the cofactor to the RNA substrate represented in pink. B, Multiple alignment between fibrillarin proteins of different origins (HUMAN, Homo sapiens; XENLA; Xenopus laevis; YEAST, Saccharomyces cerevisiae; ARCFU, Archaeoglobus fulgidus; PYRFU, Pyrococcus furiosus; and METJA, Methanocaldococcus jannaschii). Residues highlighted in blue and red (K/D/K) are absolutely conserved and correspond to the catalytic triad. The D residue in this triad is the residue mutated in this work. Bold, residues important for SAM binding. Asterisks, residues identical across all six species examined. Sequences were aligned with CLUSTAL. (TIF) [file pgen.1010012.s003.tif]

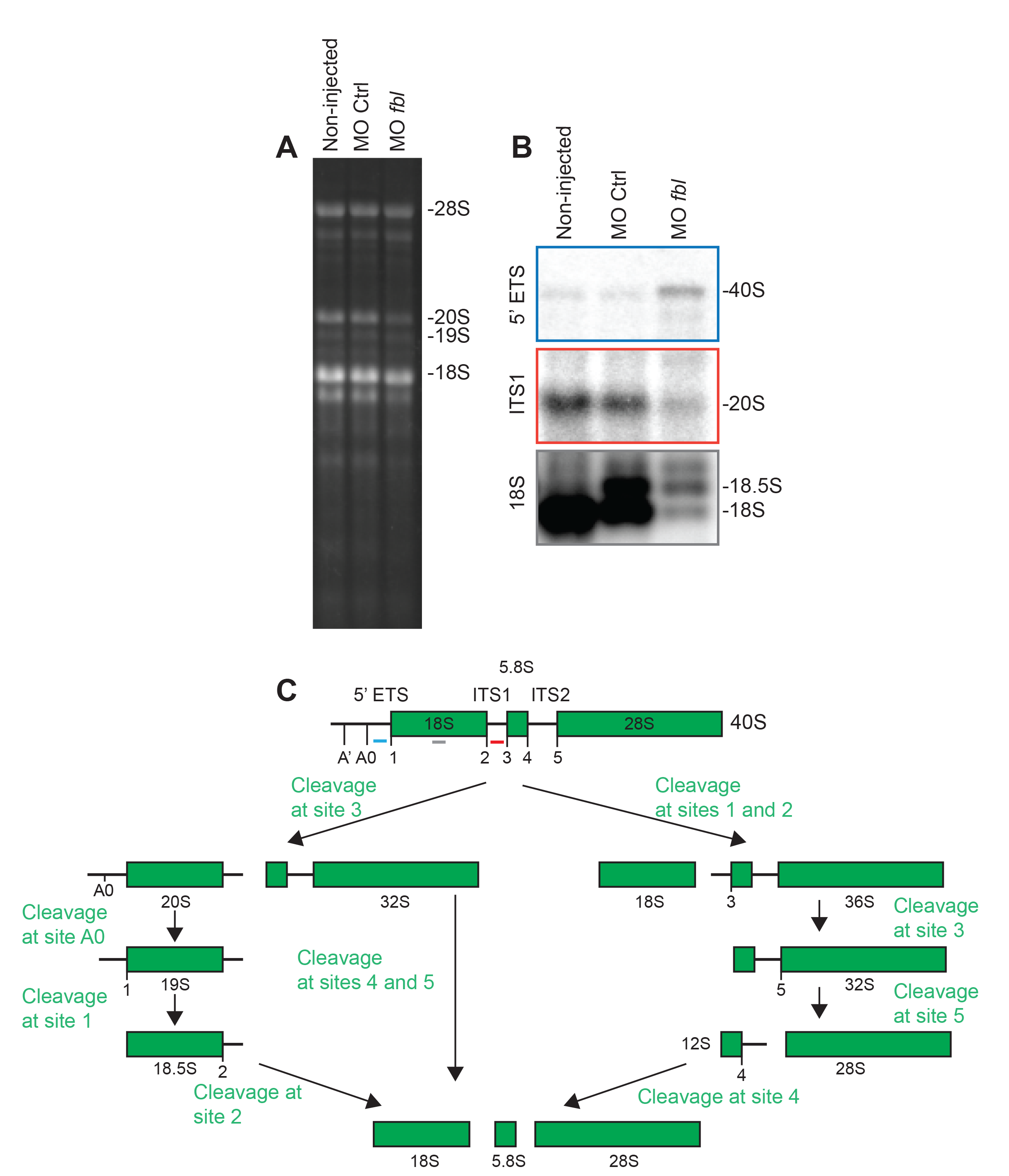

Supplement: S4 Fig — Total RNA extracted from stage 32 embryos injected into each cell at the 2-cell stage with fbl MO or with a non-targeting MO (Ctrl) was separated on denaturing agarose gel and processed for northern blotting with radioactively-labeled probes designed to detect pre-rRNA precursors. As a control, non-injected embryos were used. A, Ethidium-bromide-stained gel. Note that the mature 18S and 28S rRNAs appear as doublets, as previously described [83]. B, Northern blot analysis of pre-rRNA intermediates detected with probes specific to the 5’-ETS, the ITS1, and mature 18S rRNA (see panel C). C, Processing pathway in Xenopus [38]. Cleavage sites (A’ to 5) are indicated. The probes used in the northern blotting (panel B) are highlighted in color. (TIF) [file pgen.1010012.s004.tif]

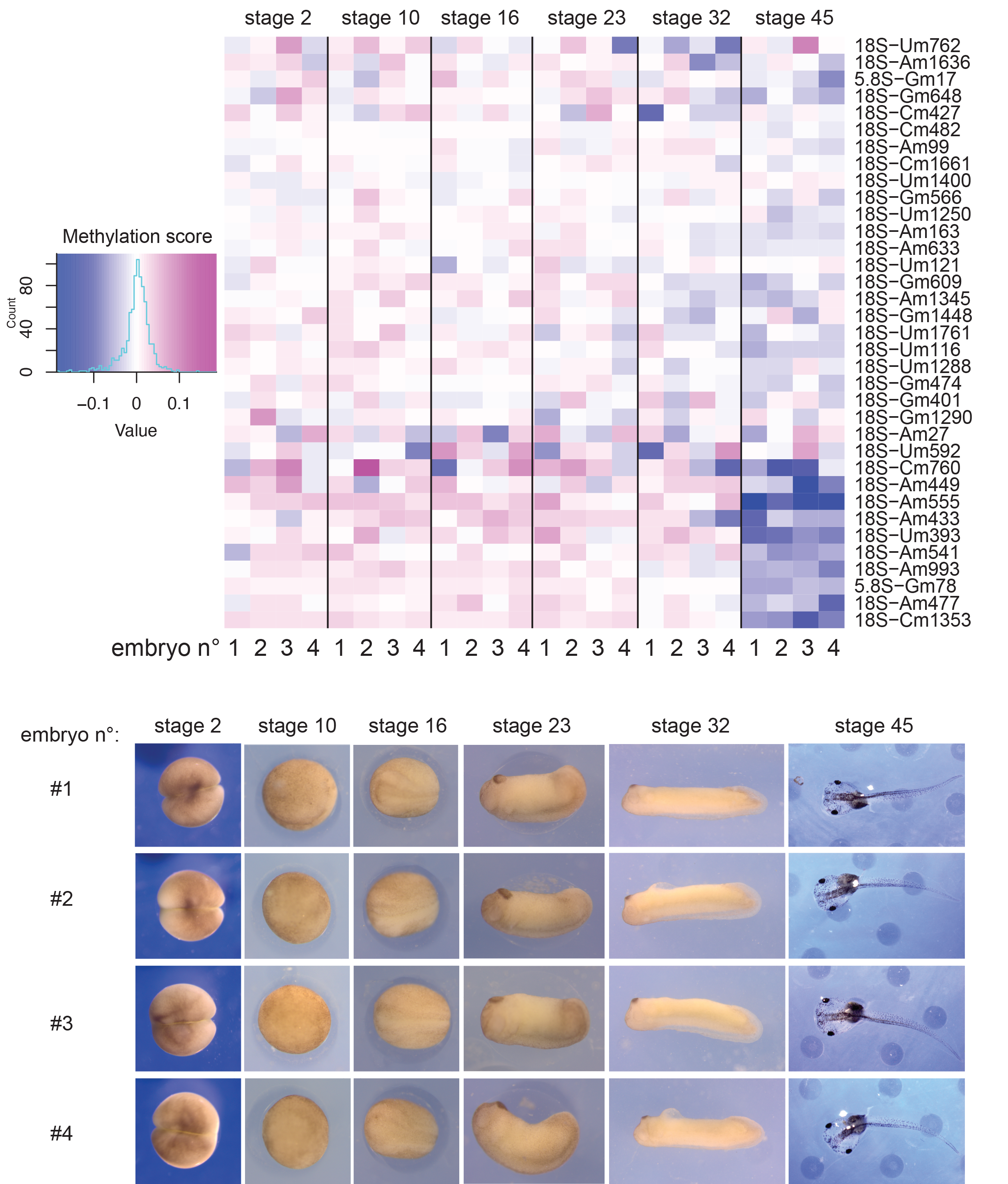

Supplement: S5 Fig — At each of the six developmental stages analyzed, four individual embryos were tested. Clustering of the RiboMethSeq analysis (here shown only for the 18S and 5.8S rRNA modifications; the same result was observed with 28S rRNA modifications) illustrates the remarkable robustness of our dataset. The staged embryos analyzed are depicted. (TIF) [file pgen.1010012.s005.tif]
