# Supplementary material for: Systematic mapping of rRNA 2’-O methylation during frog development and involvement of the methyltransferase Fibrillarin in eye and craniofacial development in Xenopus laevis
Source: PLoS Genet. 2022 Jan 18;18(1):e1010012. doi: 10.1371/journal.pgen.1010012 (PMC8797249; doi:10.1371/journal.pgen.1010012)
Supplement: Attachment — Submitted filename: Delhermite et al._Point-to-Point responses.pdf [file pgen.1010012.s012.pdf]

Point-to-point responses to the Editor's and reviewers' comments:

The reviewers raised significant concerns with the manuscript, which can possibly be addressed but will require considerable work. As reviewer 1 noted there is no mechanistic explanation for the cellular basis of the phenotype in frog fbl knockdown beyond arguing for a neural crest cell defect. Do the neural crest cells undergo apoptosis or is there a defect in proliferation and differentiation of neural crest cells. Determining where in the development process the neural crest cells are affected would go a long way toward accounting for the phenotype. Thus the disconnect between the phenotype and rDNA methylation was noted by reviewer 3 who also raised concerns about the data interpretation and recommended that conclusive proof could only come from demonstrating that rRNA is not methylated in an Fbl knockdown rescued with the Fbl-D238A mutant. Without this proof, the conclusion that rRNA through Fbl methylation is not needed for the development of NCC, but just Fbl in its role as ribosomal RNA chaperone, remains unconvincing. This would also help to address reviewer 2's comments who noted that the morphological defects were not apparently methylation dependent, but rather reflected a drastic loss of 18S rRNA. Thus the significance of any change in methylation remains unclear, and there needs to be a better connection between the two distinct components of the work.

-Comment on 'Mechanistic explanation for the cellular basis of the phenotype' (referee #1):

We now provide TUNEL assays (Fig 3, panel C).

The data shows that, upon fibrillarin depletion, cells undergo massive p53-dependent apoptosis. This is particularly the case of cells directly adjacent to the neural plate, which normally mature into NCCs and where we have shown that expression of *fbl* is more important.

-Comment on the conclusion that 'rRNA methylation is not needed to the development of NCCs' (referees #2 and #3):

We have now assessed precisely by western blot analysis the efficiency of fibrillarin depletion in the morphants. We conclude that the residual level of endogenous fibrillarin upon depletion is ~30% of the normal amount. On the basis of this new observation, we have considerably toned down our former conclusion that 'rRNA methylation is not involved in the developmental defects reported'. In fact, we no longer make any strong claim to this effect. The Results and Discussion sections have been amended accordingly.

-Comment on 'the D238A mutant' (whose importance is somehow diminished now, on the basis of the previous comment) **(referees #2 and #3)**

We now present a 3-D model of the catalytic pocket of the enzyme, showing that the mutated residue is only a few Å away from the methyl group to be transferred from the cofactor AdoMet to the substrate (Fig S3). We now also provide a better description of our choice to mutate this residue, spelling out how it was fully validated by colleagues as essential to catalysis.

Reviewer #1: Ribosomopathies are rare congenital disorders that are caused due to impaired ribosome biogenesis and in most cases affect specific tissue types. The underlying cause of ribosomopathies could be due to one of the two genetic mechanisms (1) mutations in ribosomal proteins (2) mutations in ribosome biogenesis factors. Our understanding of the factors essential for ribosome biogenesis, especially proteins required for transcription and processing of ribosomal RNAs (rRNA) remains incomplete, hence further study is needed. This manuscript explores the requirement of Fibrillarin, a rRNA processing factor, during xenopus embryonic development. The authors observe craniofacial and ocular defects in Fibrillarin morpholino knockdown embryos and identify that neural crest migration is affected due to Fibrillarin knockdown. In addition, 18S rRNA processing is impaired in the knockdown embryos, indicating that Fibrillarin is essential for rRNA processing in xenopus. Given the nature of the phenotype in fish and mouse mutants of Fibrillarin and now frog knockdown, it indicates that the function of Fibrillarin is conserved in vertebrates. However, in this manuscript there is no explanation for the cellular basis of the mechanism of the phenotype in frog fbl knockdown. Do the neural crest cells undergo apoptosis or is there a defect in proliferation and differentiation of neural crest cells?

Thank you for an overall positive assessment of our work.

In order to provide a mechanistic explanation of the cellular basis of the phenotype observed in frog upon fibrillarin knockdown, we have performed TUNEL assays (new Fig 3C).

The assays reveal that cells depleted of fibrillarin undergo massive apoptosis. The data further reveal that this elevated apoptosis is suppressed by co-depletion of p53, which agrees with the role of p53 in promoting apoptosis and with the notion that nucleolar surveillance is activated when ribosome biogenesis is compromised. Increased apoptosis was particularly obvious at the neural plate border, where we have shown *fbl* to be highly expressed and where NCCs originate.

In the second part of the paper, the authors determine previously identified and novel 2' O-Methylation sites in rRNA and identify new snoRNA. However, how this connects to the fbl mutant and the phenotype of the fbl mutant is unclear. Does the absence of Fibrillarin change specific methylation of rRNA and are those methylation marks craniofacial specific?

Referee #1 raises a very interesting question.

Regretfully, it was not possible at this stage to identify putative methylation marks specific to craniofacial development. It is possible that such marks will be identified in a follow-up work, but there is absolutely no guarantee that they exist.

Other minor suggestions:
1. Legend for Figure 2 mentions that embryos were injected with morpholinos at one cell stage. Should it be two-four cell stage instead?

Absolutely. Thank you. This was an oversight.

2. The figure quality for 2B and 2C could be improved.

We are not sure we understand this comment. We believe referee #1 must have looked at the compressed files, as on our 4K retina screens the quality of these figures is extremely high.

3. Figure 1B: It might be easier to interpret the results if the bars were grouped based on gene instead of the condition for the animal cap explant.

Thank you for the suggestion. We have produced the alternative graph (see below), but after discussion with the co-authors there was a consensus that the original display was easier to apprehend. Nonetheless, we have revised this panel by re-ordering the conditions and by adding labels to improve readability (see new Fig 1B).

Alternative version, as suggested:

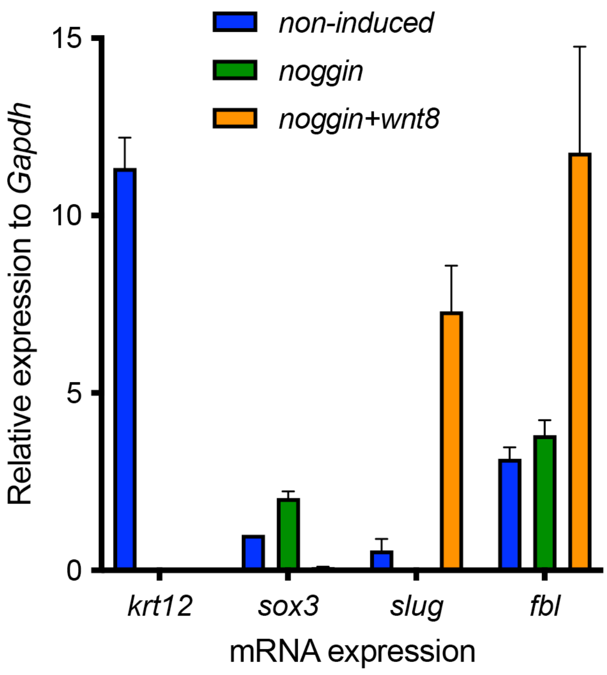

Reviewer #2: The authors set out to determine the molecular basis of inherited ribosomopathies and the potential importance of differential rRNA 2'-O-methylation during development, investigating this through depletion of the Fbl methyltransferase. The authors initially report the detection of transcripts encoding a number of ribosome synthesis factors, including FBL in embryos, with uneven distribution and enrichment in NCC cells.

FBL mRNA depletion dramatically impaired 18S rRNA maturation in Xenopus embryos, consistent with previous findings in other systems. This was associated with striking morphological defects, presumably due to altered or reduced translation. Unexpectedly, these changes were largely restored by expression of a methylation defective FLB protein. The authors use RiboMethSeq to reanalyze rRNA methylation

in detail during development. This identified 15 putative novel methylation sites, 10 of which were confirmed by primer extension. Notably several were apparently differentially modified during development.

The work is technically very good, the MS is well written, and the data on methylation will be of value. The obvious weakness in the overall structure of the MS, is that the morphological defects are apparently not methylation dependent, but rather reflect a drastic loss of 18S rRNA. In consequence, the changes in methylation are of unclear significance.

Thank you.

Comment on the 'morphological defects are apparently not methylation dependent, but rather reflect a drastic loss of 18S rRNA. In consequence, the changes in methylation are of unclear significance.'

Having now established the residual level of fibrillarin upon depletion to be ~30% of the normal amount, we no longer conclude formally that rRNA methylation is not important for normal development. We are still convinced, in agreement with referee #2, that loss of 18S rRNA production is mainly responsible for the developmental defects reported, but our new observation of residual expression certainly makes possible a contribution of methylation. We have revised our model accordingly. Incidentally, our revised model brings further coherence to our work.

Overall, the MS presents interesting and useful data on the spatial distribution of ribosome synthesis factors, morphological effects of reduced ribosome synthesis during development and changes in modification. Better linking these two sections would significantly strengthen the MS.

Thank you.

Specific comments:

1. It was unexpected that the morphological changes were largely restored by expression of a methylation defective FLB protein. This indicates that residual FBL expression on knockdown is sufficient to support some, presumably adequate, level of methylation that supports ribosome function.

Referee #2 is absolutely correct about this, and we are extremely grateful for this comment, which has led us to establish the level of residual endogenous fibrillarin in the morphants as well as the expression levels of the rescue constructs.

Using an antibody specific to fibrillarin, it was possible to show that in the morphants, there was about 30% residual endogenous protein (Fig 2, panel D, lane 2).

Since our original rescue constructs were Flag-tagged (and we have now changed the labels throughout the manuscript and figures to make this clear), it was also possible

to check by western blotting that they are well expressed, using an anti-Flag antibody (Fig 2, panel D, lanes 3 and 4).

This analysis revealed that both rescue constructs, now renamed Flag-Fbl-wt and Flag-Fbl-D238A, were well expressed.

This further shows that expression of the rescue constructs increased the global amount of fibrillarin to 40-50%, which also provides an explanation of the partial morphological rescues (see Fig 2E, half-normal-sized branchial arches were formed, green arrowheads) (and see below).

It would be useful to indicate what, if any, defects are seen in cells complemented by expression of the catalytic mutant, since these are likely due to a lack of methylation. Their description and discussion would potentially link the tow section of the paper. It would also be helpful to indicate the residual level of wt FBL in the complemented animals.

First part of the comment: It was not possible technically to address defects in individual cells complemented by expression of the catalytic mutant.

Second part of the comment: As mentioned above, the residual levels of fibrillarin in the morphants and rescued animals have now been established (see Fig 2, panel D).

We completely agree with referee #2 that this information was very important to establish. This information has allowed us to refine our model (residual expression of endogenous protein) and provides an explanation for the partial rescues.

Ideally, RiboMethSeq might indicate what level of methylation is sufficient to support close to normal development. However, this may not be feasible at present.

We agree that this would be very useful information to be gained. The problem is that such an experiment is extremely challenging to perform successfully at this stage, for several reasons. The principal reason is the persistent presence of modified ribosomes of maternal origin, which are abundant and stable during the first embryonic stages inspected. Another complication is the residual expression of endogenous wild-type fibrillarin in the morphants.

2: Figs. 1, S1 and associated text: The text has several sections with statements like "…suggesting that Fbl plays an important role at these locations". As FBL is believed to be essential for ribosome synthesis in all cells, this statement seems inappropriate. It would be better to make the interpretation more explicit. Presumably what the authors intend to conclude is something like: elevated FBL transcript abundance may be associated with increased FBL protein synthesis. If FBL is limiting for ribosome synthesis, this increased production might alter the rate or pathway of pre-rRNA packaging, processing or modification. This point is raised in the Discussion, but it would be better to be explicit from the start.

We absolutely agree. Thank you. The text has been amended accordingly.

Reviewer #3:

This article consists of two, somehow distinct, messages. First, the expression of the protein fibrillarin is quantified at different stages during the development of Xenopus laevis. Fbl knockdown is found to cause morphological defects, which are attributed to defects in the development of neural crest cells. Second, the authors quantify site-specific methylations of ribosomal RNA during development, identify additional fifteen positions and observe a (slight) increase in hypomethylation in the late stages of development. The manuscript provides some new data relative to Xenopus laevis, which are found to be consistent with the literature reports on other species. The level of novelty is limited. Nevertheless the data are worth publishing.

Thank you. Note that what we assessed at different stages by performing WISH were the levels of *fbl* mRNA (and not protein).

All in all, I have doubts on the demonstration that the defects in NCC observed upon Fbl knockdown are not due to the Fbl methylation activity.

This comment echoes one of referee #2's concerns, which we have addressed above.

Having established the residual level of endogenous fibrillarin after depletion as 30% of the normal amount of the protein, we no longer conclude that methylation plays no role in early embryonic development. We have fully revised our model accordingly.

First, the rescue effect with the methylation incompetent mutant is far from being complete;

As referee #3 will agree, partial suppression of severe morphological alterations is not rare in such rescue assays.

We completely agree that expression of our rescue constructs, and this is true of both the Flag-Fbl-wt and Flag-Fbl-D238A constructs (Fig 2), only partly suppresses the developmental defects - see for example the restoration of only half-normal-sized branchial arches (Fig 2E).

As discussed above in response to referee #2, we believe our new western blot analyses presented in Fig 2, panel D offer a good explanation for these partial morphological rescues, the overall level of fibrillarin reaching 40-50% when one combines the residual endogenous and rescue proteins.

second, this mutant has been demonstrated to be methylation incompetent in archaea fibrillarin but not in eukaryotic fibrillarin.

This particular aspect has become much less crucial than it may have been before, since we no longer make any formal claim that methylation is not involved in the observed developmental defects (see comments above).

Nonetheless, in the revised text, we explain much better why we have selected residue D238 for mutagenesis and why we are convinced there is no reason to believe that

the protein harboring the D238A mutation is active in methylation. Again, considering our new interpretation of the data, this point has clearly become secondary.

Even if the sequence conservation of fibrillarin is high across species, the sequence conservation of the N-terminal domain of the Nop proteins is not; this may cause subtle rearrangements in the enzyme that change the mutant activity relationships.

As can be seen in the new Fig S3, the mutated residue (in red) is far from the N-terminal extension. In fact, it is truly buried within the catalytic pocket of the enzyme, being located within ~3 Å of the methyl group to be transferred from the AdoMet cofactor to the RNA substrate. The team that established the 3-D structure of the enzyme has concluded not only that this residue is essential for catalysis (involving a general acid-base mechanism) but also that it is likely important to provide an electrostatic surface for facilitating AdoMet binding (doi: 10.1038/nsb905). The role in catalysis was later validated experimentally by the same team using an extremely powerful *in vitro* assay (doi: 10.1074/jbc.M406209200). The second reference was already in our manuscript and we have now added the first one as well with an additional description.

A much more convincing proof would be demonstrating that the rRNA is indeed not methylated in the Fbl knockdown rescued with the Fbl-D238A mutant. Without this proof, the conclusion that rRNA through Fbl methylation is not needed for the development of NCC, but just Fbl in its role as ribosomal RNA chaperone, remains unconvincing.

We agree with referee #3 that direct proof that the rRNA is indeed not methylated in the Fbl knockdown rescued with the Fbl-D238A mutant would have been ideal. Unfortunately, this was not achievable because of 1) the 30% residual wild-type endogenous fibrillarin present in the rescued embryos, and 2) the persistent presence of modified ribosomes of maternal origin. Of course, on this basis, and taking fully on board the comments of both referee #2 and referee #3, we have completely revised our initial model, and we no longer formally conclude that methylation is not important for development.
