# Supplementary material for: Systematic mapping of rRNA 2’-O methylation during frog development and involvement of the methyltransferase Fibrillarin in eye and craniofacial development in Xenopus laevis
Source: PLoS Genet. 2022 Jan 18;18(1):e1010012. doi: 10.1371/journal.pgen.1010012 (PMC8797249; doi:10.1371/journal.pgen.1010012)
Supplement: Attachment — Submitted filename: Response to Reviewers_15Nov2021.pdf [file pgen.1010012.s013.pdf]

**Point-to-point responses on the second decision letter:**
While the reviewers recognize the improvements you have made to the original manuscript there are two key issues that they note as remaining outstanding.

The first is that the biological insights of the manuscript remain somewhat limited.

We are really confused by this comment.

We feel this is rather unusual to receive a comment on biological significance at this stage considering the manuscript has been with *PLoS Genetics* for several months already.

We discuss below why we feel strongly such a comment is incorrect.

We would like to highlight that *PLoS Genetics* has published several ribosome biogenesis-related works performed in *Xenopus* recently (for example from the Baserga and Khokha's Lab). We are presenting a similar amount of information of very high quality with respect to the developmental part of our work. Specifically, we demonstrate that the box C/D snoRNA-associated methyltransferase Fibrillarin is required for early embryogenesis in *Xenopus laevis*, and, in particular, for neural crest cells survival with a very fine description of a facial dysmorphy phenotype reminiscent of neurocristopathies and a redistribution analysis of prototypic developmental markers. Please have a close look at the quality of our WISH, complementation analysis etc.

But in addition, we also report the full repertoire of ribosomal RNA 2'-O methylation in *Xenopus laevis*, confirming previously mapped sites, identifying new sites, confirming new sites by use of an orthogonal approach (low dNTP primer extension), and revealing for the first time in this organism the existence of hypomethylation at specific positions. We also report a comprehensive bioinformatic screening of the *Xenopus laevis* transcriptome leading to the assignment of individual SNORD guides for each modified position. We feel important to highlight also that our RNA modification mapping was performed on four individual embryos at six embryonic stages, in a resolution which has not been achieved before. We therefore feel our work is highly significant biologically.

The second is the lack on integration between the phenotypic data resulting from fibrillarin MO knockdown and the absence of an effect on rDNA methlyation.

Firstly, we have not addressed DNA methylation but RNA methylation. This mistake was already present in the first feedback we received (first Decision Letter), but then we thought it was simply an oversight. We realize now it is mentioned five times in the front paragraph of the report, which is confusing.

We believe the comment on lack of integration is overly emphasized. Considering Fibrillarin is a methyltransferase active on ribosomal RNA, to us, it is perfectly logical to study in the second section of our work the sugar methylation landscape across development. To take this comment into account and to improve the transition, we included a short liaison paragraph.

There is also an absence of co-localization of cell death and other parameters with neural crest cells as is commonly presented in zebrafish or mouse models of ribosomopathies.

Although this comment is not very detailed, we assume it refers to a proliferation marker such as phospho-histone 3.

Answer: We have shown in our revision that cells are undergoing massive apoptosis using a TUNEL assay, which was very finely controlled (p53 suppression; and DMRT5 technical control). Having shown the cells are dying, we feel it is not necessary to show they don't proliferate, as this is implied.

I recognize that the authors have toned down their statement that fibrillarin is required for rDNA methylation or that defects in rDNA methylation underpin the phenotype.

Thank you. Indeed, we have carefully taken on board all the previous comments. Again, we have characterized rRNA methylation (not rDNA methylation) and concluded it is not responsible for the phenotypes.

However, the fact that the fibrillarin morphant phenotype can be rescued with a methylase dead fibrillarin construct suggests that the fibrillarin morphant phenotype is not caused by defects rDNA methylation.

Absolutely agree! And that's exactly what we wrote in our manuscript. To make it even more clear, we have re-emphasized our conclusion.

The flow of results in the papers moves from phenotype to methylation to phenotype to preRNA processing, to ribosome biogenesis and p53 rescue which makes the paper difficult to follow. It might make more sense for the methylase dead fibrillarin rescue to come after the evaluation of rDNA methylation sites such the rescue would allow the authors to conclude the phenotype is not due to deficient methylation, but rather deficient ribosome biogenesis.

Thank you for the suggestion, we appreciate it. There are many ways to tell a story and a choice of a storyline remains somehow a personal one.

We have discussed the suggestion among co-authors. We believe it is logical and important to present the wild-type and the catalytic rescues “side-by-side” as they directly control each other.

The authors could further demonstrate this by testing what happens to the rRNA species post fibrillarin rescue. Is processing and are the levels return to normal. This might help explain why only a 10-20% increase in fibrillarin levels from the 30% baseline of the morphants is sufficient to elicit a substantial rescue.

We are thankful for the suggestion. This is a new experiment, which was not requested before at the first revision round. The result of this experiment is totally implied in our current results. Indeed, since we have shown that the methylation function of Fibrillarin is not important for early development (our rescue assay), this implies it is its role in processing which is.

To understand this fully, it is important to keep in mind how fibrillarin works: during the early steps of ribosomal subunit assembly, fibrillarin binds to nascent pre-rRNA transcripts. This early binding step must occur for subunit biogenesis to proceed faithfully (RNA processing) and, importantly, it is independent of its catalytic activity in methylation (see e.g. https://doi.org/10.1016/j.molcel.2019.08.014). We have explained this further in our revised manuscript and included this reference.

**Reviewer's Responses to Questions**

**Comments to the Authors:**

**Reviewer #1:** While the manuscript has definitely improved from the first submission, here are two points of concern:

1. Since the cellular basis for the phenotype of fbl mutant is apoptosis, the defect is lack of NCC survival rather than maturation.

Of course, it is not a problem to introduce this semantic change. We have amended our manuscript accordingly at all instances.

2. In addition, since there is no direct connection between fbl and 2’ O-Methylation data, the paper seems like two different stories. One about Fibrillarin’s requirement in craniofacial development and the other about rRNA modification. I am not convinced that the two data belong together.

We respectfully disagree with this comment. How could one section dealing with the role of a protein in early development, and the other characterizing its rRNA substrates systematically and quantitatively for the first time across early development not be related? To us, it is completely logical that these two sections belong together.

**Reviewer #2:** The MS has been improved and my specific comments have been suitably addressed. I am happy to support publication of the revised MS.

Thank you.
